# On the shape of cicada’s wing leading-edge cross section

**DOI:** 10.1038/s41598-021-87504-4

**Published:** 2021-04-08

**Authors:** Rachel M. Starkweather, Svetlana V. Poroseva, David T. Hanson

**Affiliations:** 1 Department of Mechanical Engineering, University of New Mexico, Albuquerque, NM USA; 2 Department of Biology, University of New Mexico, Albuquerque, NM USA

**Keywords:** Aerospace engineering, Engineering, Biological physics

## Abstract

An important role that the leading-edge cross-section shape plays in the wing flight performance is well known in aeronautics. However, little is known about the shape of the leading-edge cross section of an insect’s wing and its contribution to remarkable qualities of insect flight. In this paper, we reveal, in the first time, the shape of the leading-edge cross section of a cicada’s wing and analyze its variability along the wing. We also identify and quantify similarities in characteristic dimensions of this shape in the wings of three different cicada species.

## Introduction

Insects’ wings appear simple and fragile. Yet, thin, translucent, and essentially weightless compared to the body it carries, the wing works admirably from an aerodynamic perspective. Many aspects of insect flight have previously been investigated^1–21^*,* but little is known about the shape of the insect’s wing leading-edge cross section. On the other hand, it is well known that the leading-edge cross-section shape plays an important role in controlling the flow separation and transition to turbulence in all wings, including those of insects^16,22–28^, and in all aspects of multidisciplinary aircraft design.

These considerations motivated the current study, which goal is to determine the shape of the leading-edge cross section of the forewings (hereafter, wings) of a common North American insect, a cicada, and how this shape varies along the wing. We chose a cicada for the study based on our previous research^29–31^*,* where we demonstrated aerodynamic benefits of implementing some of the features of cicada’s wing and body into designs of the rotor blade and the proprotor-nacelle assembly.

Previously, cross sections of veins in the forewings of the *Manduca sexta* species^18,19^ and the Monarch butterflies^20,21^ were analyzed, with images and dimensions being provided in^18–20^ for the *Manduca sexta* hawkmoth and the Monarch butterfly, respectively. However, the leading-edge cross-section area was not well resolved in either of the previous works, and its cross section was assumed as circular for the measurements and the analyses, even though it is clearly non-circular and non-elliptical^18^. Detailed structural analyses of the wings were conducted in^18–21^ and of aerodynamic performance of the Monarch butterfly wing with rectangular and circular vein models in^20,21^. No effects of the leading-edge cross-section shape on the insect’s wing aerodynamics were previously investigated, and no realistic shape is currently available, to our best knowledge. Thus, the purpose of the current paper is to provide a broad community of professionals with the leading-edge cross-section shape of cicada’s wings to enable future studies of its aerodynamic effects. We also hope that our research with motivate similar studies on the leading edge of the wings of other insects to facilitate their comparative analysis and stimulate collection of data of the dimensions of insects’ bodies and wings along with the insect’s masses by a broader community of entomologists and enthusiasts to advance our understanding of insect flight. The methodology presented in the paper is applicable to investigating the wings of any insect.

## Results

**Figure 1:**
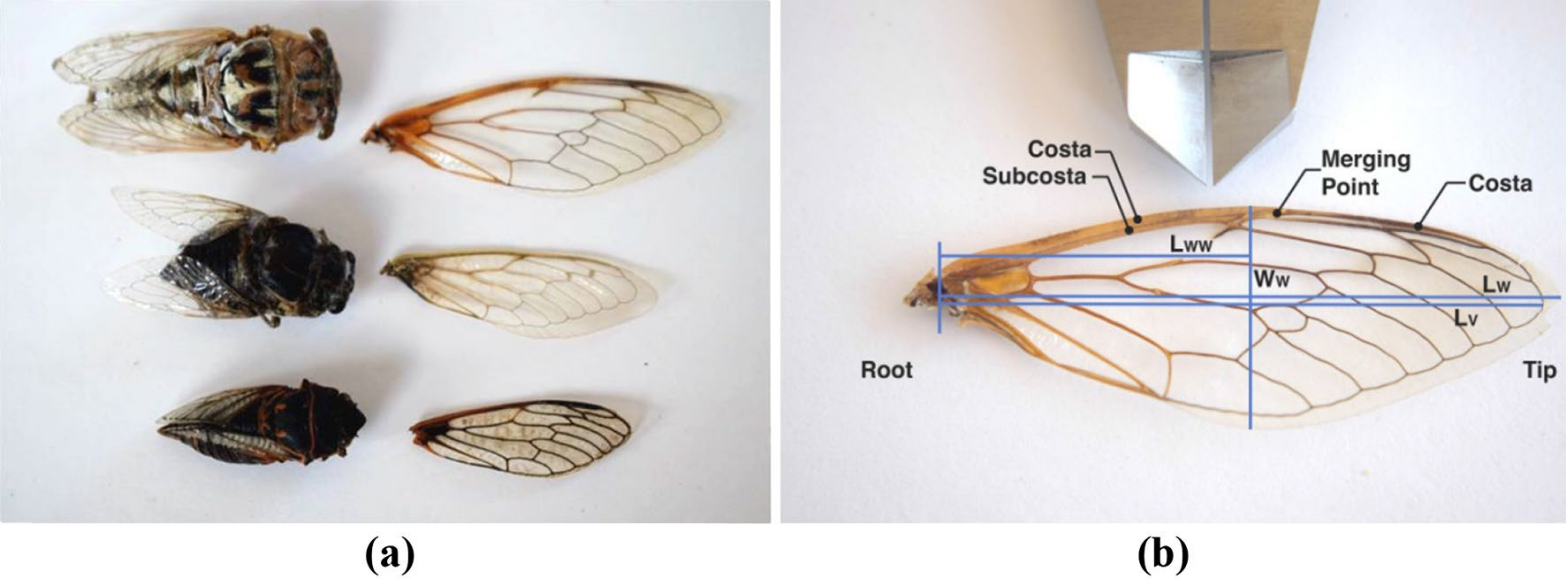
Cicadas considered in the study with one of their removed forewings displayed to the right. Images: (**a**) the species used in the study from top to bottom: *Megatibicen dealbatus, Cacama valvata*, and *Tibicen duryi,* (**b**) the characteristic parts and dimensions of a cicada’s wing.

Specimen of three cicada species: *Megatibicen dealbatus* (commonly known as a Plains Cicada or the Plains Harvest-Fly), *Cacama valvata* (also a Cactus Dodger), and *Tibicen duryi* (Fig. 1a) were collected for the study from June to September 2019 in various regions of the state of New Mexico, U.S.A. The collection ascertained that wings of different cicadas and cicada species shared common features in their leading edge. Hereafter, the species will be referred to as Cicada 1, Cicada 2, and Cicada 3, respectively.

We conducted a visual inspection of specimen from a pool of insects to identify a set representative of its species. We then selected one insect per species from the sets and sectioned and examined their wings over the course of several months. Whereas slicing more wings may reveal more similarities and differences among the wings of different species, this is not the goal of our study. We are looking for obvious similarities, which would be statistically impossible events as accidental mutations in randomly picked specimen from 3 different species.

Figure 1b shows the left wing of Cicada 1 as an example with characteristic lengths, which will be used for the data analysis:

- the wing length, $L_{w}$, defined as the distance between the middle point of the wing root (the body-wing joint location) and the wing tip (location farthest from the body);
- the distance between the wing root and the farthest vein projected on the axis aligned with the wing length (hereafter, the wing length axis, *x*), $L_{v}$;
- the wing width, $W_{w}$, defined as the largest distance between the leading and rear (trailing) edges in the direction normal to the wing length axis; and
- the distance between the wing root middle point and the location of the wing width measurement, $L_{ww}$.

Table 1 and Supplementary Fig. 1 online provide values of these lengths for individual wings. For comparison, the insect bodies and their dimensions are also given in Table 1 and Supplementary Fig. S2 online. The body dimensions, such as width, $W_{b}$, and length, $L_{b}$, are those for the body planform. The body length is defined as the largest distance between the body tip and rear, and the body width is the largest distance between the body planform edges in the direction normal to the axis aligned with the body length (hereafter, the body length axis).

**Table 1:** Cicada wing and body dimensions.

Dimension	Cicada 1	Cicada 2	Cicada 3
Body length, *L*_*b*_, mm	33.7	26.6	27.1
Body width, *W*_*b*_, mm	16.2	13.2	10.5
Wing length, *L*_*w*_, mm	44.6	35.3	27.4
Wing width, *W*_*w*_, mm	16	11.6	9.6
Costa and subcosta merging point location, *L*_*m*_, mm	24.5	19.3	16.0
Distance from the wing root to the farthest vein, *L*_*v*_, mm	43.3	32.3	27.1
Distance from the wing root to the location of the wing width measurement, *L*_*ww*_, mm	22.3	19.0	16.9
Body volume, *V*_*b*_, mm^3^	4630.8	2426.8	1564.4
Wing area, *S*_*w*_, mm^2^	560.5	321.6	206.6

In the wing part closest to the insect body, the wing leading edge is composed of two veins: costa and subcosta (Fig. 1b). Moving away from the body towards the wing tip, the two veins merge into the single vein, costa. Absorption of the subcosta into the costa is typical for cicada wings^7^*.* In the study, the location of the costa and subcosta merging point, $L_{m}$, (Fig. 1b) was determined in the three wings (Table 1).

**Figure 2:**
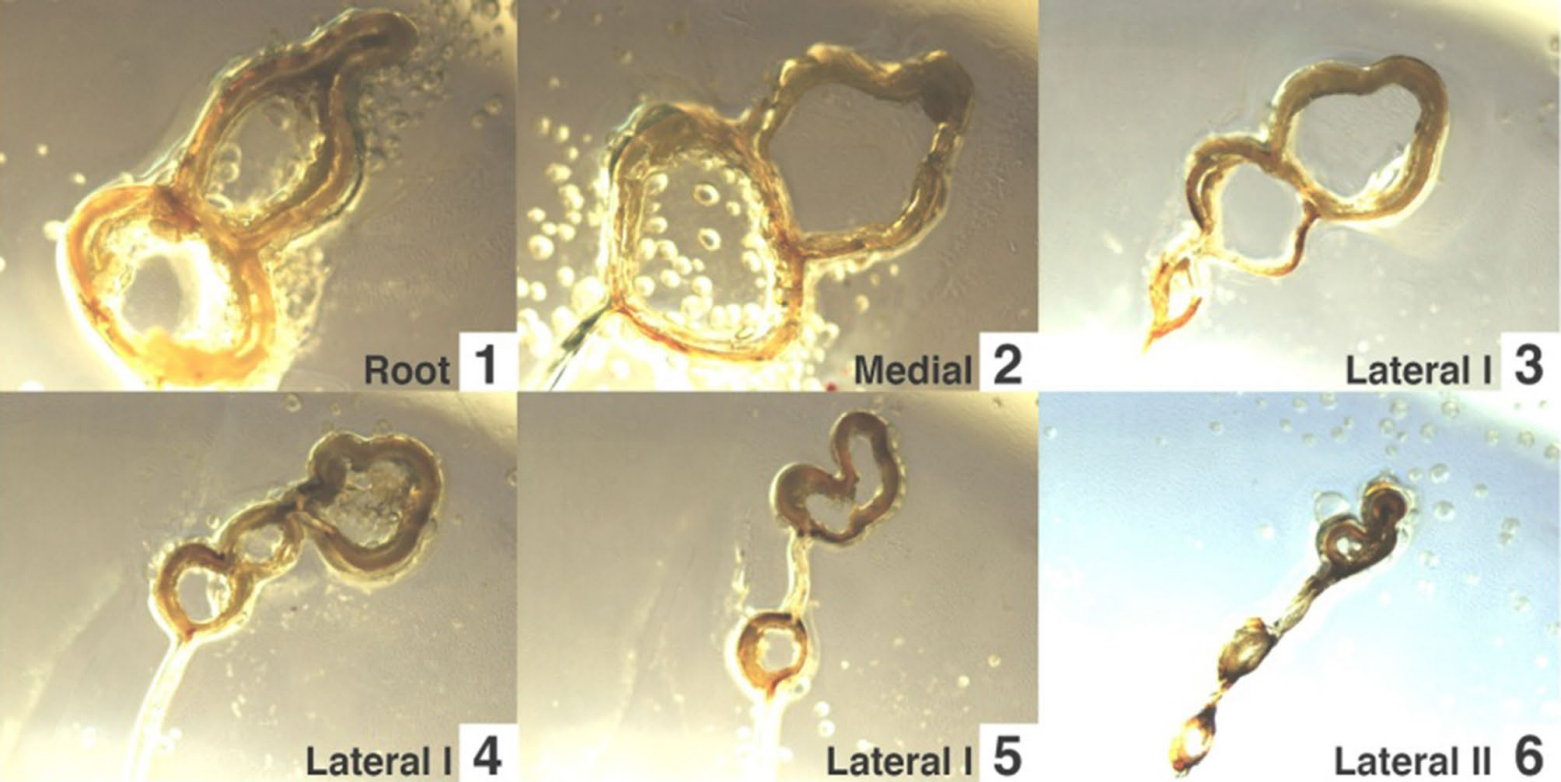
Cross sections of the Cicada 1 wing leading edge at different locations.

We obtained images of the leading edge cross sections and measured characteristic dimensions of the costa and the subcosta at different locations along the leading edge (Supplementary Tables S1–S4 online). Figure 2 demonstrates evolution of the leading-edge cross-section shape along the Cicada 1 wing. Examples for the wings of Cicada 2 and 3 are shown in Supplementary Fig. S4 online. In the figures, labels from 1 to 6 correspond to the samples in the direction from the wing root to the wing tip.

**Figure 3:**
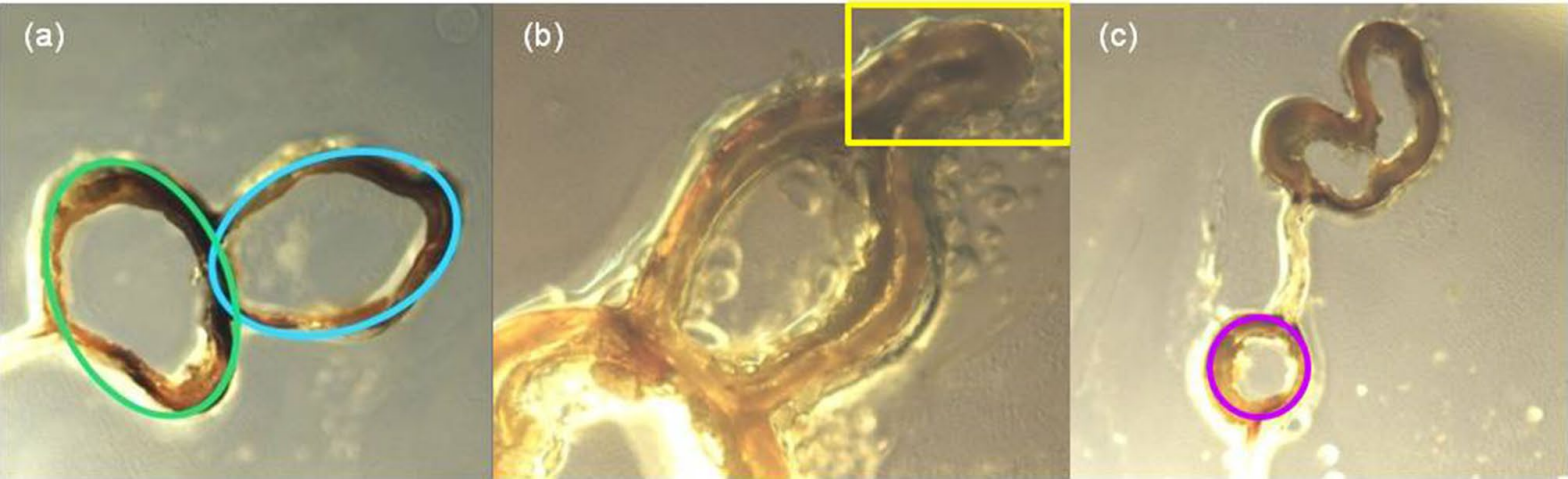
The Cicada 1 wing leading-edge cross section. Images: (**a**) a cross section with the costa and the subcosta approximated by ellipses (blue and green, respectively), (**b**) a cross section with the costa tip located within the area marked by yellow line, (**c**) a cross section with the marked internal vein (purple circle).

The three wings exhibited similarities. One was the lack of circularity in the costa and subcosta profiles along the wing. The subcosta shape can be approximated by an ellipse (green line in Fig. 3a), with its major axis normal to the wing membrane and its minor axis in the wing membrane plane at all locations where the images were available.

The shape of the costa cross section is the most perplexing one, and it is also common in the wings of different cicada species. This feature is best described as an ellipse (blue line in Fig. 3a), which major axis is aligned with the wing membrane plane and its top is pinched. Hereafter, the “pinched” costa ellipse top is called the costa tip and is located within the area marked by yellow line in Fig. 3b. The costa tip is rigid and persists along the leading edge. Although measurements of the tip dimensions were not possible everywhere along the wing leading edge, it appears that the tip tends to become wider and shorter in the direction from the wing root to the costa and subcosta merging point and then becomes again more pointed towards the wing tip.

Since the cross sections of veins inside the wing membrane tend to be circular (purple circle in Fig. 3c), the shape of the cicada wing leading-edge cross section is likely to be a response to aerodynamic forces experienced by the wing during flight. It is interesting to note that this shape is very different from that of the leading edge of airfoils, which are cross sections of man-made wings and blades. The airfoil leading edge usually has a simple smooth shape^32–38^ that can be approximated by a single circle or ellipse similar to the costa shape (blue line in Fig. 3a) without its tip.

We measured dimensions of the costa, subcosta, and costa tip along the leading edge (where possible) in the three wings. These are the total height of the leading edge, $H_{t}$, heights of the costa, $H_{c}$, and the costa tip, $H_{ct}$, widths of the costa, costa tip, and subcosta: $W_{c}$, $W_{ct}$, and $W_{s}$. The subcosta height, $H_{s}$, was calculated as the difference between $H_{t}$ and $H_{c}$.

Data were also collected for the wall thicknesses of the costa and subcosta: $T_{c}$ and $T_{s}$. Additional information on the variation of the wall thickness along the wing can be obtained by considering the difference of the vein outer and inner perimeters. For this reason, outer and inner perimeters were also measured for the costa and subcosta. The perimeter data were converged to equivalent diameters,$D_{co}$ and $D_{ci}$ for the costa and $D_{so}$ and $D_{si}$ for the subcosta, with indices *o* and *i* corresponding to “outer” and “inner” values, where *D* is defined as $D = P/\pi$ (here, *P* is any perimeter). Figure S5 shows the measured dimensions and Supplementary Tables S1–S4 online provide the data for these parameters.

Epistemic uncertainty in all measurements presented in this paper is due to natural asymmetries present in organic bodies and those caused by the process of separation of wings from the bodies affecting the wing root conditions, body drying, wing rehydration and processing. However, this uncertainty does not prevent extracting the common features that can be recognized in the three wings of different cicada species including the shape of the wing leading-edge cross section. Such features would be statistically impossible events as accidental mutations in randomly picked specimen from 3 different species. The following section provides discussion of our findings.

## Discussion

In aerodynamics, non-dimensional parameters are normally used. This is the approach adopted in the current study as well. Several such parameters were identified in the study and are presented in Table 2. They were selected based on their numerical values in individual cicada wings, which deviate less than $10\%$ of their average values for the three cicadas.

**Table 2:** Ratios with the close values in the three cicadas wings.

Ratios	Cicada 1	Cicada 2	Cicada 3	Average value	Max deviation, %
$W_{w} /L_{w}$	0.36	0.33	0.35	0.35	5.7
$V_{b} /S_{w}$	8.26*h*_*w*_	7.50*h*_*w*_	7.57*h*_*w*_	7.80*h*_*w*_	5.9
$L_{m} /L_{w}$	0.55	0.55	0.58	0.56	3.4
$L_{v} /L_{w}$	0.97	0.92	0.99	0.96	4.2
$W_{w} /W_{b}$	0.48	0.50	0.39	0.45	6.5
$\max S_{t} /S_{w}$	0.0141	0.0129	0.0129	0.0133	6

In the table, all deviation values are absolute. The cicada body volume, $V_{b}$, and the wing area, $S_{w}$, were approximated as those of an ellipsoid and an ellipse, respectively (Table 1), with their major and minor axes being the body/wing length and width; $h_{w}$ is the nominal thickness of the wing membrane introduced for the unit’s consistency and assumed to be the same for all cicadas. The wing leading-edge cross-section area, $S_{t}$, was calculated as the sum of the two veins areas: $S_{t} = S_{c} + S_{s}$. The vein area was approximated as by an ellipse using the vein height and width as the lengths of the major and minor axes, respectively.

We considered the evolution of the leading edge cross section dimensions along the wing with respect to the leading-edge projection on the wing length axis, *x*. All dimensions and respective locations along *x* were normalized by the wing length. The wing was partitioned into several sections along the axis: Root, Medial, Lateral (Lateral I and Lateral II for Cicada 1), and Tip, in the direction from the wing root to the wing tip (Supplementary Fig. S2 online), to facilitate the discussion. The costa and subcosta merging point was located in the wing Lateral (Lateral I for Cicada 1) section.

**Figure 4:**
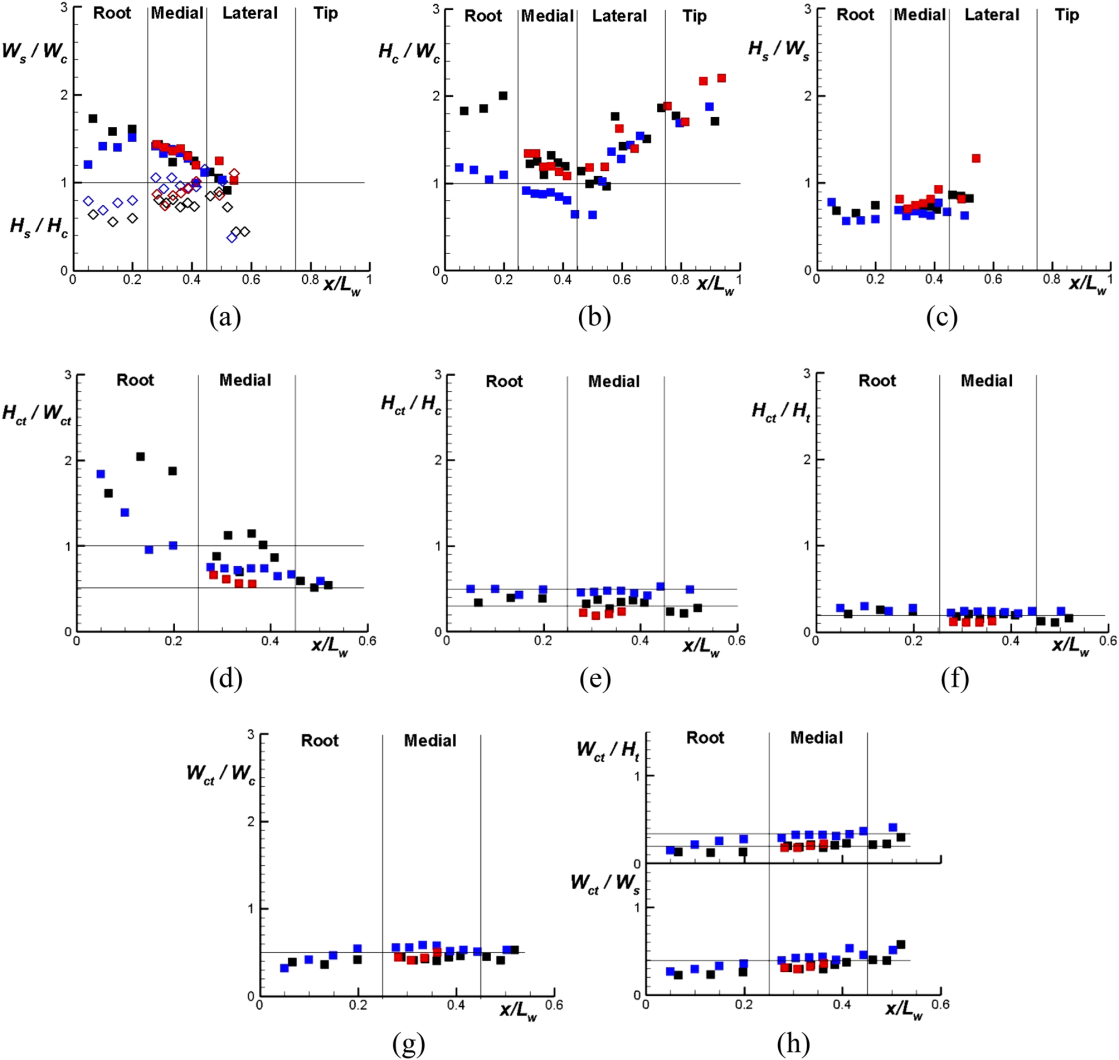
Ratios between the leading edge characteristic heights and widths along the wing in the direction from the wing root ($x/L_{w} = 0$) to its tip ($x/L_{w} = 1$).The boundaries of the wing Root, Medial, Lateral, and Tip sections as shown correspond to $x/L_{w} = 0.25,0.45,$ and 0.75, approximate values for the three cicadas. Color scheme: black – Cicada 1, blue – Cicada 2, red – Cicada 3. Symbols in (**a**): open—$H_{s} /H_{c}$, closed—$W_{s} /W_{c}$.

The heights and widths of the costa and the subcosta vary differently in the wing Root section of different cicadas (Fig. 4a). Nevertheless, general tendencies can be recognized even in this section; the costa height is larger and the costa width is smaller than the corresponding dimensions of the subcosta (Fig. 4a). The differences between the costa and subcosta heights and widths tend to reduce away from the insect body, with the width changing more than the height in the wing Medial section. In fact, the costa and subcosta heights are close to each other in this wing section.

In the proximity of the costa and subcosta merging point, the widths of the costa and the subcosta are approximately equal to each other and so are their heights. That is, the veins tend to restore their circularity to merge. The width value, where $W_{c} = W_{s}$, can be estimated as 4.0% of the wing length for the three cicadas. The height value, where $H_{c} = H_{s}$, varies more for the three wings, in the range between 3.0% to 4.3% of the wing length.

After the costa and the subcosta have merged, the costa height and width continue to reduce, but the reduction slows down in the wing Tip section. At the location closest to the wing tip, values of the two parameters are in the ranges of 1.5–3.0% and 1–1.3% of the wing length for the height and width, respectively.

Although the costa width and height reduce individually towards the wing tip, their ratio grows from the costa and subcosta merging point towards the wing tip (Fig. 4b). This is a common feature in the cicada wings. Similar tendency is observed when moving from the merging point to the insect body: $H_{c} /W_{c}$ grows again, but somewhat slower (Fig. 4b). In the Lateral and Tip parts of the wing, the ratio $H_{c} /W_{c}$ and dependence on $x/L_{w}$ are close for the three cicadas. In the Root and Medial section, the ratio variation with $x/L_{w}$ is similar for the three cicadas, but there is a noticeable scatter in the values for the three wings.

In contrast, the ratio $H_{s} /W_{s}$ for the subcosta changes little before the subcosta is absorbed into the costa (Fig. 4c), with the ratio close in the three cicada wings. The data also demonstrate that in the subcosta, the width is generally larger than the height everywhere except in the proximity of the costa and subcosta merging point. The reverse tendency is observed for the costa, that is, its height is generally larger than the width everywhere except again in the proximity of the costa and subcosta merging point.

Overall, it is worth repeating that the circularity of the two veins’ cross sections appears to be of particular importance for the two veins to merge. The elliptic shapes and their orientation are of importance when moving away from the merging point in both directions: toward the wing tip and toward the insect body.

The ratio of the tip height to its width, $H_{ct} /W_{ct}$, reduces in the direction from the insect body toward the costa and subcosta merging point (Fig. 4d), with the tip height twice as large as the tip width close to the insect body, but the tip width twice as large as the height in the merging point proximity.

The ratio of the costa tip and costa heights, $H_{ct} /H_{c}$, varies little along the wing. There is a small reduction in its value in the wing Root part in the direction toward the costa and subcosta merging point, which is similar in the two wings (no such data are available for the third wing). In the Medial part, the ratio value can be approximated as constant for the three cicadas in the range between $0.3L_{w}$ and $0.5L_{w}$ (Fig. 4e). Less scatter is observed when the costa tip height is normalized by the total leading edge height: $H_{ct} /H_{t}$, with the ratio average value of $0.2L_{w}$ in both the Root and Medial parts of the three wings (Fig. 4f).

When considering data for the costa tip width, results for the ratio $W_{ct} /W_{c}$ (Fig. 4g) are similar to those for the ratios $W_{ct} /W_{s}$ and $W_{ct} /H_{t}$ (Fig. 4h). That is, the costa tip width with respect to these parameters slightly increases through the wing Root section, but changes little in the wing Medial part. When $W_{ct}$ is normalized by the costa and subcosta widths, $W_{ct} /W_{c}$ and $W_{ct} /W_{s}$, the values were found to be common for the three cicadas: 0.5 and 0.4, respectively.

All widths and heights used in Fig. 4 are also shown in Supplementary Fig. S6 online normalized by the wing length to complement information above. When looking at the behavior of the costa tip width and height (open black circles in Supplementary Fig. S6 online), we notice that in the wing Medial section, both parameters change little. The average value of $W_{ct} /L_{w}$ is 0.019 $\pm 0.003$ and characteristic of all wings in this wing section. The same cannot be said about $H_{ct} /L_{w}$, whose values differ noticeably in the three wings.

We considered many other ratios that include the costa, subcosta and costa tip widths and heights, but found them less useful in highlighting common tendencies in the wings of different cicadas.

The evolution of the costa and subcosta wall thicknesses along the wing is similar and so are their values (Supplementary Fig. S7 online). The larger the wing dimensions, the larger the costa and subcosta wall thicknesses in the wing Root section. In the Medial and Lateral sections, the costa wall thickness changes little among the three cicadas, in the range of 0.001 $L_{w}$ – 0.004 $L_{w}$, with the average value of 0.003 $L_{w}$. The data for the subcosta wall thickness in this wing section are more scattered. The difference between the vein outer and inner perimeters (represented by the equivalent diameters): $\Delta D_{c} = D_{co} - D_{ci}$ and $\Delta D_{s} = D_{so} - D_{si}$ for the costa and the subcosta, respectively (Supplementary Fig. S7 online), provides additional insight into the variation of the costa and subcosta wall thicknesses along the wing. These values are less scattered than those for the wall thicknesses and they generally support observations based on the data of direct wall thickness measurement. That is, the costa and subcosta wall thicknesses reduce towards the wing tip. Outside the wing Root section, the difference in the subcosta and costa wall thicknesses is not significant. In addition, the perimeter data show that outside the wing Root section, the differences in wall thicknesses among the three wings are not significant and tend to converge to the same value for the costa and the subcosta in all wings in the proximity of the costa and subcosta merging point, which can be estimated as 0.005 $L_{w}$. Since the costa and subcosta shapes in this location also tend to become circular, the relation between $\Delta D = \Delta D_{c} = \Delta D_{s} = 0.005L_{w}$ and the wall thickness $T = T_{c} = T_{s}$ can be established as $T = \Delta D/2 = 0.0025L_{w}$, which is in close agreement with values of $T_{c}$ and $T_{s}$ close to the costa and subcosta merging point.

## Summary

The study revealed in the first time the shape of the leading-edge cross section of a cicada’s wing, analyzed its variability along the wing, measured characteristic dimensions of the shape at various locations along the wing, and identified multiple similarities in this shape among the wings of three different cicada species. The presented results will enable future research of aerodynamic effects of the leading-edge cross-section shape of a cicada’s wing. We also hope that our research will (1) motivate similar studies on the leading edge of the wings of other insects to facilitate their comparative analysis and (2) stimulate collection of data of the dimensions of insects’ bodies and wings along with their masses by a broader community of entomologists and enthusiasts to advance our understanding of insect flight.

## Supplementary Information

Supplementary Information.

## Data Availability

Data are available in the main text and in the Supplementary Materials online.
