# Supplementary material for: On the shape of cicada’s wing leading-edge cross section
Source: Sci Rep. 2021 Apr 8;11:7763. doi: 10.1038/s41598-021-87504-4 (PMC8032777; doi:10.1038/s41598-021-87504-4)
Supplement: Supplementary file 1 — Supplementary Information. [file 41598_2021_87504_MOESM1_ESM.docx]

Supplementary Materials for

On the Shape of Cicada’s Wing Leading-Edge Cross Section

Rachel M. Starkweather^1^, Svetlana V. Poroseva^1*^, David T. Hanson^2^

Correspondence to: poroseva@unm.edu

Materials and Methods

Materials

Materials for Wing Rehydration:

- 4-in. inner diameter plastic container with lid
- Damp paper towels
- Lavender Essential Oils
- Tree Tea Essential Oils
- 4x4 metal sieve, 3-in. diameter
- Forceps
- Latex Gloves
- Dried Cicada wings

Materials for Cryostat Preparation:

- Rehydrated Cicada wings
- Digital camera
- Digital metric caliper
- Latex gloves
- Forceps
- Utility blade
- Cutting board
- 4-5 disposable base molds per wing (0.94 in. x 0.94 in.)
- 4 oz. bottle optimal cutting temperature compound (OCT)
- Ultra-fine point permanent marker
- Insulated gloves
- Safety goggles
- Lab coat
- Insulated bucket rated for LN_2_
- Small metal stand, 2 in. tall
- 5-liter dewar
- 3 liters liquid nitrogen (LN_2_)
- 1 colored pencil
- 4-5 small plastic sample bags per wing
- 1 box disposable low-profile microtome blades
- Small paint brushes
- Tweezers
- Microscope slide case
- 100 pre-cleaned microscope slides per wing

Equipment for sectioning and image processing:

- Thermo Scientific Cryostat HM550
- Zeiss Axioskop 2 mot plus Fluorescence Motorized Microscope with AxioCam HRc
- USB 3.0 flash drive
- HP ENVY x360 touch-screen
- Fiji image process package

Methods

Three species of cicada were collected from various regions of in the state of New Mexico. The *Tibicen duryi* were collected in the desert brush from northeastern New Mexico near Taos in June 2019. The *Cacama valvatas* were gathered in the foothills of the Sandia mountains in Albuquerque, New Mexico among the brush and cacti during July 2019. The *Megatibicen dealbatus* were collected in Albuquerque, New Mexico in August and September 2019 along the Rio Grande River. Upon collection, the cicadas were air-dried to prevent molding and rotting.

In order for sectioning to take place, the wings were rehydrated to mitigate crumbling. Preliminary attempts to cut dry wings were unsuccessful regardless a choice of tools and methods used for cutting and a presence (or absence) of the wing coating with various supporting materials.

To rehydrate the wings, damp paper towels were placed at the bottom of a 4-in. diameter plastic container filling up half the container. Five drops each of lavender and tea tree essential oils were added to the paper towels to prevent the growth of bacteria. A 4x4 metal sieve was then placed inverted with the mesh side up on top of the paper towels to serve as a platform to elevate the wings above the paper towels; this allowed for circulation of humid air around the wings. The dried wings were placed on the mesh sieve using forceps and latex gloves. The container was secured with a lid, and the wings were left for four days to fully rehydrate. After this process, the wings regained their flexibility and malleability allowing for preparation for sectioning. In entomology, this process of rehydration is a common method for preparing, mounting, and staging insects [38]*.*

Images were taken of the wings before their sectioning. A metric caliper was set to 0.05 millimeters and was photographed in images with each rehydrated cicada wing to be used as a scale for the wing size.

For sectioning, each wing was placed on the cutting board using latex gloves and forceps. Using the utility blade, the wings were cut in four or five pieces, depending on the size of the wing (Supplementary Fig. S3 online). The first cut of the wings was made by finding the location where the costa and subcosta visibly began to merge and a third vein became visible running parallel to the leading edge. A cut was made at approximately 4 mm before this point closer to the region where the wing connects to the insect body. Once this cut was made, the two wings halves were cut again approximately in half. For Cicada 1, the distal portion of the wing was cut in three parts so each section of the large wing would fit in the disposable plastic cryostat molds. Based on the wing cutting procedure, the wing sections were named: Root, Medial, Lateral (Lateral I and Lateral II for Cicada 1), and Tip in the direction from the insect body to the wing tip, with the costa and subcosta merging point being in the Lateral section of the Cicadas 2 and 3 wings and in the Lateral I section of the Cicada 1 wing.

The cut wings were documented with photographs.

Disposable base molds were marked with a permanent market noting the cicada type and the section of the wing to be placed in the mold. The molds were filled halfway with optimal cutting temperature compound (OCT). Using the forceps, the wing sections were each placed in an individual mold on top of the OCT with the leading-edge veins near the top of the mold. The molds were filled completely with OCT allowing for the wing sections to be suspended in the compound.

The metal stand was placed in the bottom of the insulated bucket with the opening downwards. Using the lab coat, insulated gloves, and safety goggles, liquid nitrogen was poured from the dewar into bottom of the bucket until the level was approximately a quarter inch below the top of the channel. While still wearing personal protective equipment, the forceps were used to place the molds on top of the c-channel. After the OCT turn from translucent to opaque, the forceps were used to retrieve the molds. The samples were marked with a colored pencil to note the location of the leading edge and the desired cutting direction. The samples were removed from the molds and placed in labeled laboratory sample bags.

A Thermo Scientific Cryostat HM550 set to -20 ºC was used to examine the cross sections of the wing. A lab coat and latex gloves were worn. First, a sample was adhered in the proper orientation to the cryostat specimen disk using OCT and allowed to harden in the temperature-controlled chamber. Once opaque, the specimen disk was loaded and secured in the mounting area using the screws to fasten its position and angle. A low-profile microtome blade was placed in the stage blade holder, and the blade cover was removed. Using the external stage controls, the specimen was moved to be in close proximity with the blade.

The specimen cutting increment was set to 100 microns. The excess OCT material was cut from the specimen by making repetitive passes using the stage hand-crank until the cicada wing veins were visible in the specimen. Once the wing was reached, the stage hand-crank was used to cut a single slice at a time to be collected on a slide. The sliced specimen was transported to the microscope slide using tweezers and brushes. Several specimen samples were place on each slide. The cicada type, the wing section, and the slide number (for tracking the order the sample were retrieved) were marked on the slide with a permanent marker and placed in the slide box. This process of collecting samples was repeated until the wing specimen was completely sectioned. The cryostat was cleaned, the blade replaced, a new specimen was loaded, and the process repeated.

Once all the samples were collected from each wing specimen, the slides were examined under a Zeiss Axioskop 2 mot plus Fluorescence Motorized Microscope with AxioCam HRc at 5X magnification. The cross section samples that were undamaged with visible leading edge cross sections were photographed using the microscope’s digital camera. A scale was added to the images for 5X magnification. The same scale was used for all images of each sample. The images were added to a USB 3.0 flash drive.

After all the images were taken, FIJI (previously Imagej) image processing software [39] was downloaded onto a laptop with a touch screen. In FIJI, the pixel count was set to the scale of the image using the 5X scale. Using the touch screen and the measurement settings, several characteristic dimensions of the two veins were measure. The total height of the leading edge, $H_{t}$, was measured from the tip of the costa to the base of the subcosta, where the wing membrane began (Supplementary Fig. S5a online). The costa height, $H_{c}$, was taken from the costa tip to the middle point of the costa-subcosta intersection (Supplementary Fig. S5b online). The subcosta height, $H_{s}$, was calculated as the difference between $H_{t}$ and $H_{c}$. Both the costa and subcosta widths, $W_{c}$ and $W_{s}$, were measured at their widest point between their outer edges (Supplementary Figs. S5b and S5c online). After the costa and subcosta converge into one vein, the overall cross section height and the costa width were used as the cross section dimensions. The costa tip height, $H_{ct}$, was determined as the shortest distance betwen the costa tip and the imaginary line between two points of the costa curvature (Supplementary Fig. S5d online). The costa tip width, $W_{ct}$, was measured as the largest distance between the costa tip outer edges in the direction normal to the costa height (Supplementary Fig. S5d online). Data was also collected for the wall thicknesses of the costa and subcosta: $T_{c}$and $T_{s}$, at approximately 90 degress assuming clockwise movement from the tip of the costa (Supplementary Fig. S5e online). The location was chosen as the one representative of the average wall thickness after visual inspection of all images.The outer and inner perimeters were measured for the costa and subcosta by tracing the vein walls exculding any reminents inside the veins (Supplementary Figs. S5e-S5h online).

The process was repeated for the each sample image. The data is provided in Supplementary Tables S1-S4 online.


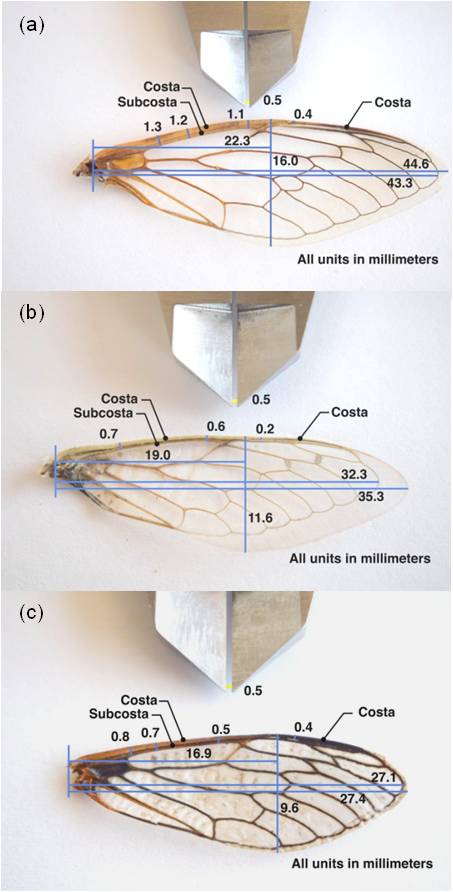


Supplementary Fig. S1.

Dimensions of cicada wings. Images: (a) Cicada 1, (b) Cicada 2, (c) Cicada 3. Blue vertical lines show locations of the wing root and width. Blue horizontal lines show distances from the wing root to the locations of the wing width measurement, the vein furthermost from the wing root, and the wing tip.


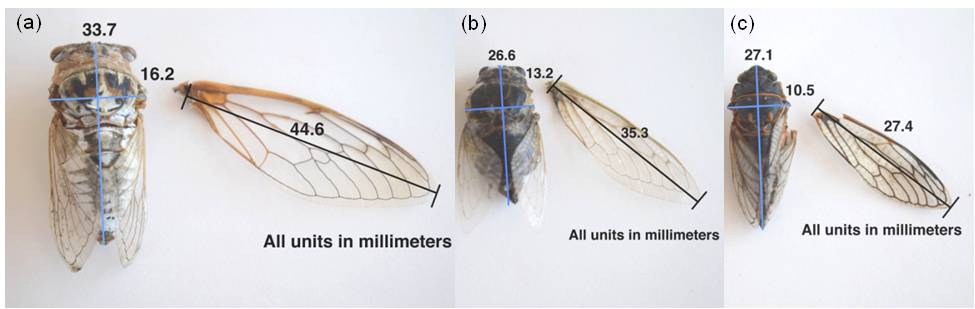


Supplementary Fig. S2.

Dimensions of cicada bodies in comparison with the wing length. Images: (a) Cicada 1, (b) Cicada 2, and (c) Cicada 3. Lines: blue - the body length and width, black – the wing length.


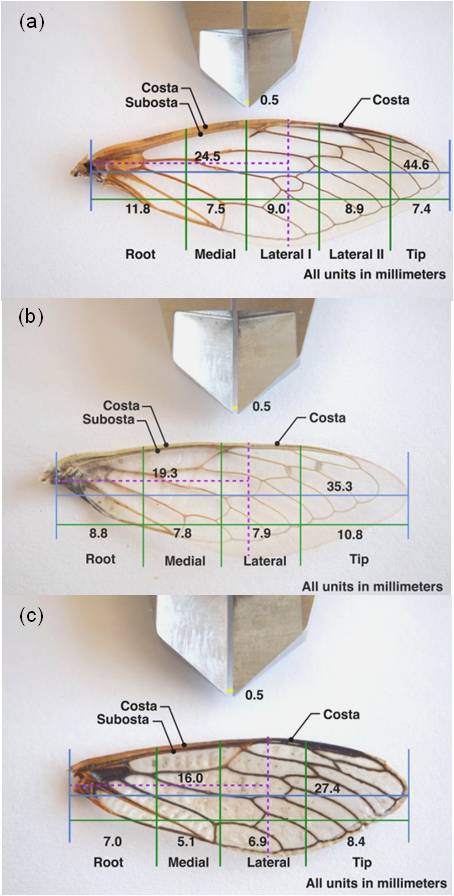


Supplementary Fig. S3.

Wing sections and the location of the costa and subcosta merging point: (a) Cicada 1, (b) Cicada 2, and (c) Cicada 3. Lines: blue - the wing length, green - boundaries of the wing sections and their lengths projected on the wing length axis, violet - the projection of the costa and subcosta merging point on the wing length axis and the distance to this location from the wing root.


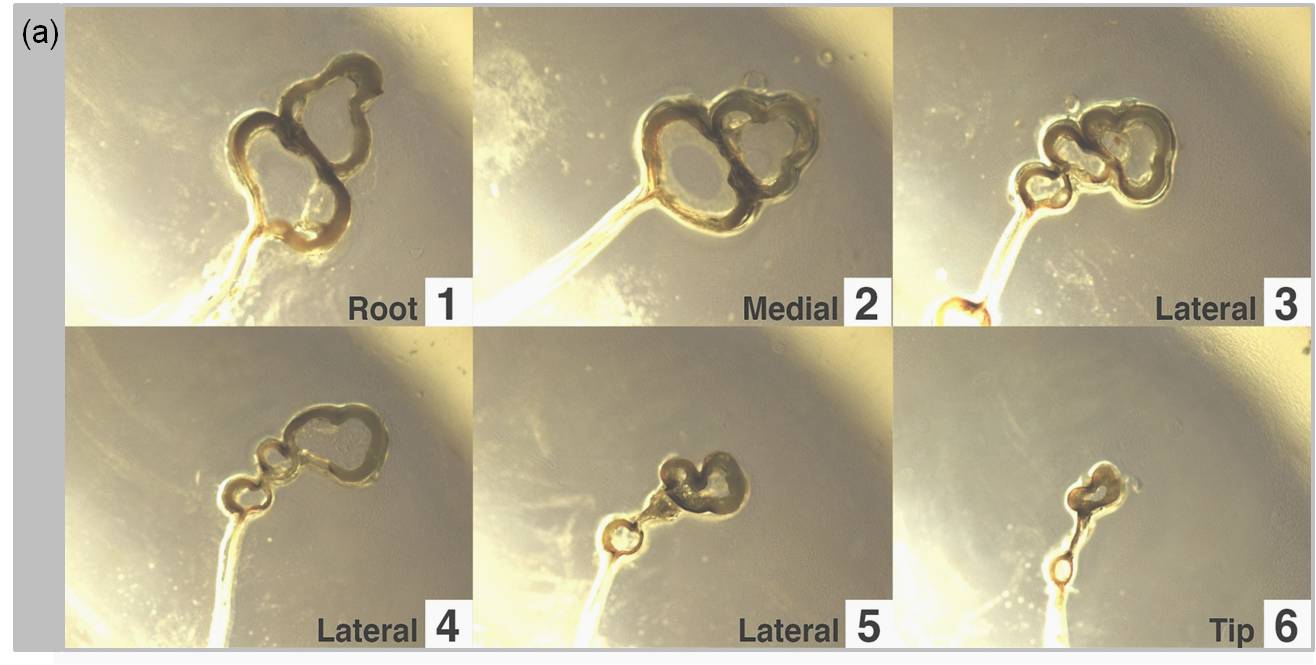


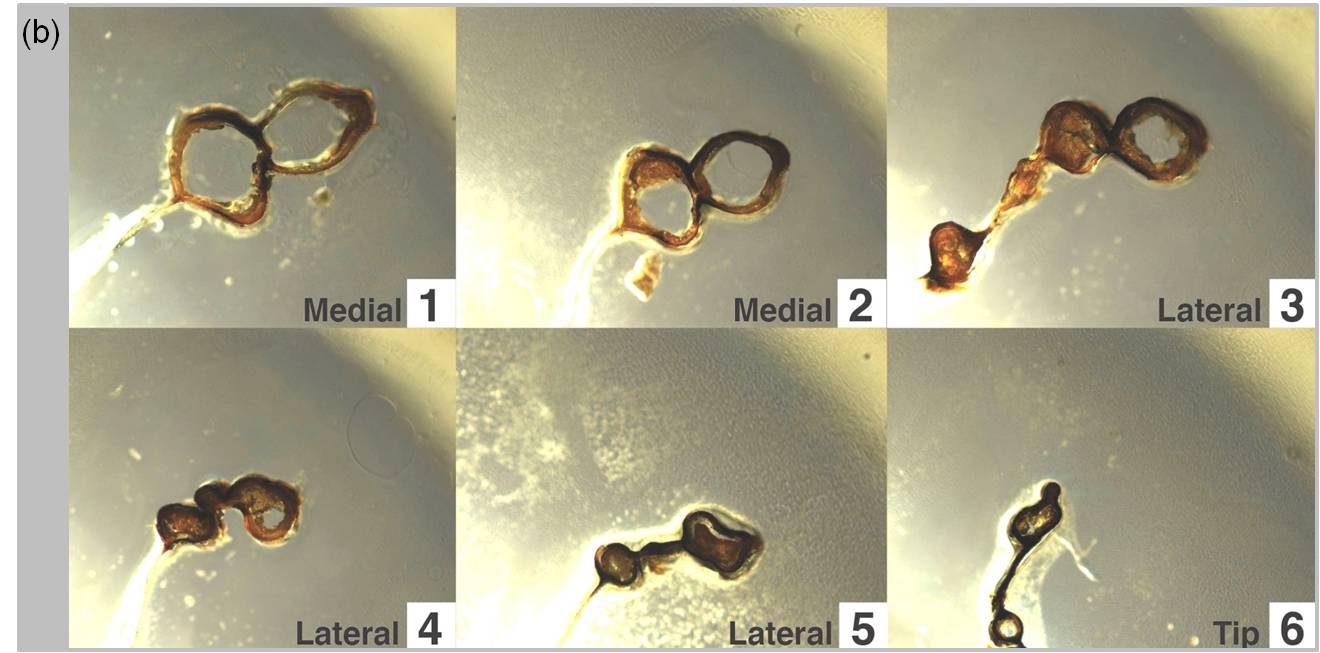


Supplementary Fig. S4.

Cross sections of the wing leading edge: (a) Cicada 2 and (b) Cicada 3. Labels from 1 to 6 in the figures correspond to the samples in the direction from the wing root to the wing tip.

**
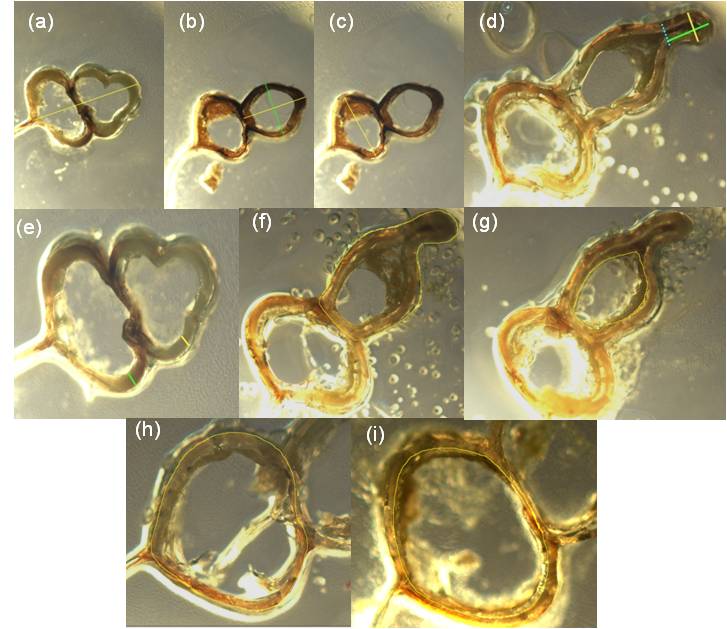
**

**Supplementary Fig. S5.**

Measured dimensions of the wing cross sections shown by color lines. Yellow lines: (a) the total height of the cross section, (b) the costa height , (c) the subcosta width, (d) the costa tip width, (e) the costa wall thickness, (f) the costa outer perimeter, (g) the costa inner perimeter, (h) the subcosta outer perimeter, (i) the subcosat inner perimeter. Green lines: (b) the costa width, (d) the costa tip height, (e) the subcosta wall thickness. Blue line: (d) the imaginary costa tip boundary.


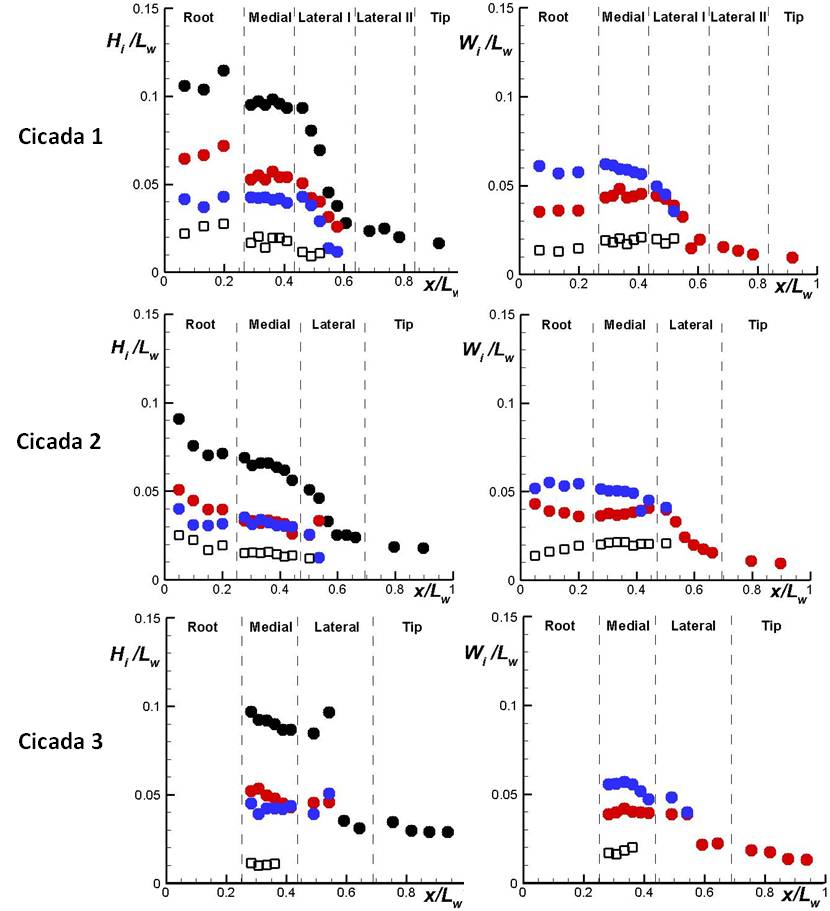


Supplementary Fig. S6.

Variation of the characteristic heights and widths of the wing cross section along the wing leading edge projected on the wing length axis *x.* Locations $x/L_{w}=0$ and 1 correspond to the wing root and tip, respectively. Symbols: – the total cross section, – the costa, – the subcosta, – the costa tip.


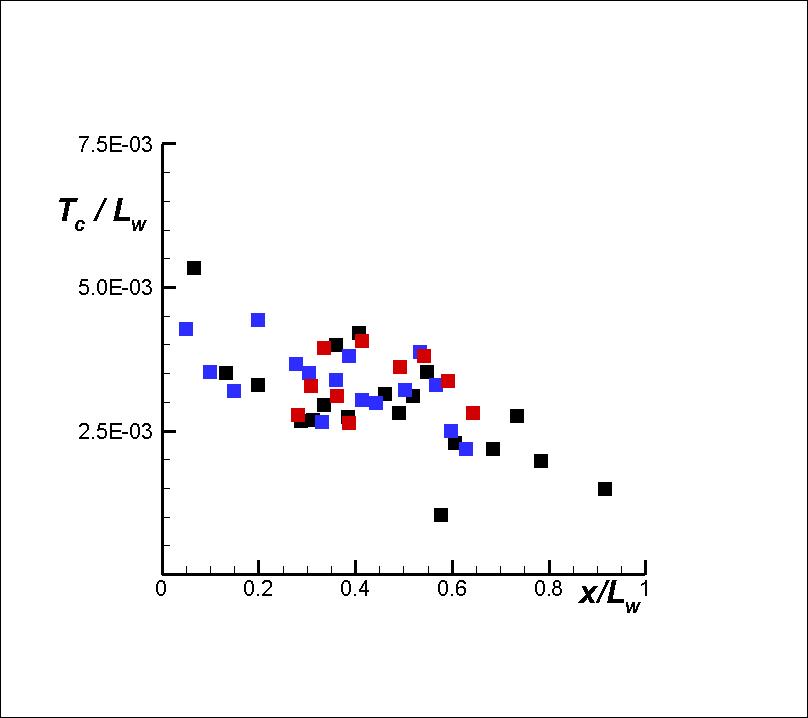

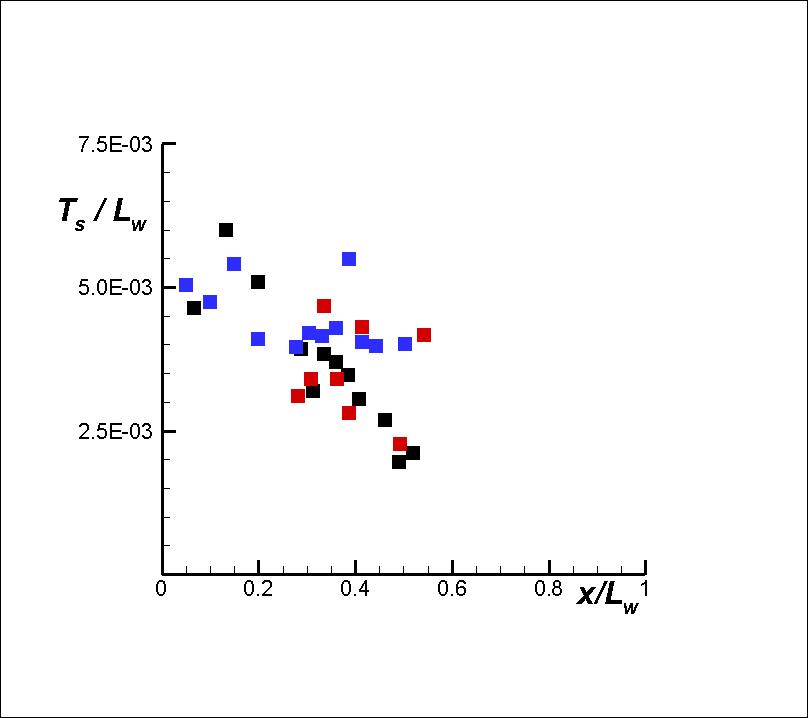


(a)


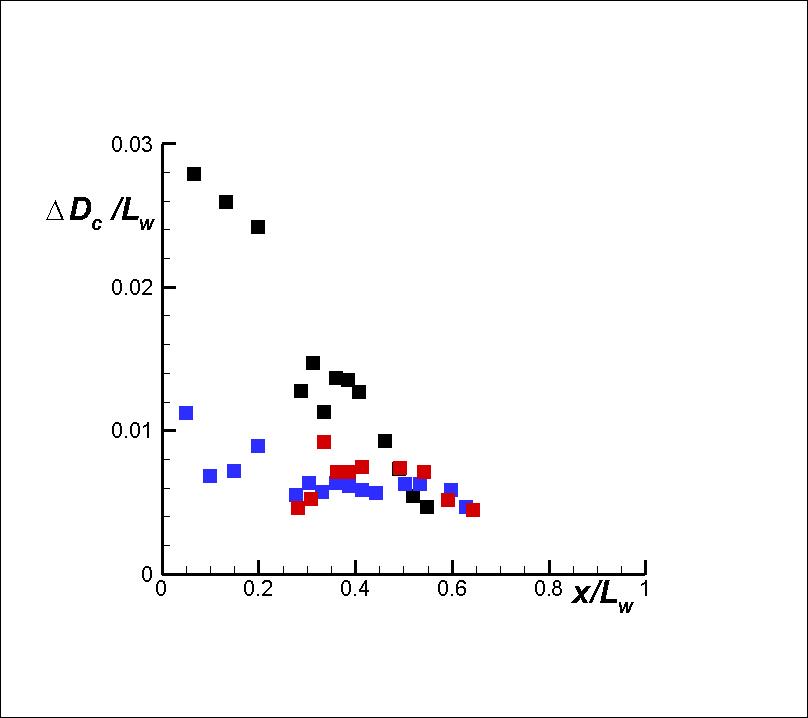

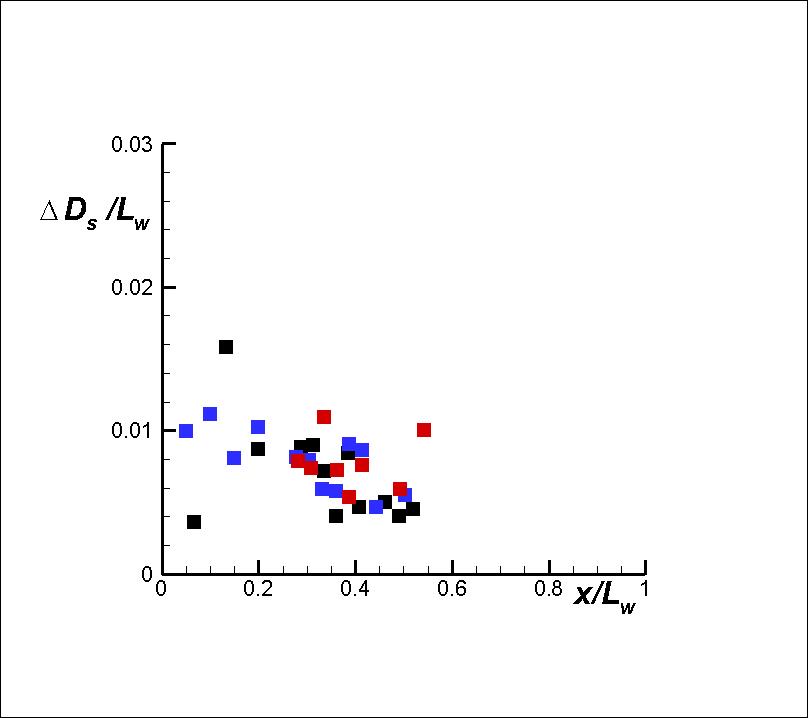


(b)

Supplementary Fig. S7.

Variation of the costa and subcosta wall thicknesses along the wing leading edge projected on the wing length axis. Images: (a) the costa, (b) the subcosta*.* Symbols: – Cicada 1, – Cicada 2, – Cicada 3.

| Wing section | Sample  number | Cross section total height,  *H_t_*, *μm* | Costa height, *H_c_*,*μm* | Costa tip height, *H_ct_*, *μm* | Costa width, *W_c_*, *μm* | Costa tip width, *W_ct_*, *μm* | Subcosta width, *W_s_*, *μm* |
| --- | --- | --- | --- | --- | --- | --- | --- |
| Root | R1 | 4730 | 2883 | 2721 | 1579 | 609 | 2721 |
| Root | R2 | 4636 | 2978 | 2536 | 1607 | 577 | 2536 |
| Root | R3 | 5117 | 3204 | 2571 | 1600 | 660 | 2571 |
| Medial | M1 | 4247 | 2351 | 2769 | 1929 | 855 | 2769 |
| Medial | M2 | 4344 | 2456 | 2744 | 1970 | 808 | 2744 |
| Medial | M3 | 4244 | 2351 | 2644 | 2148 | 907 | 2644 |
| Medial | M4 | 4388 | 2546 | 2632 | 1926 | 770 | 2632 |
| Medial | M5 | 4281 | 2414 | 2562 | 1959 | 870 | 2562 |
| Medial | M6 | 4168 | 2413 | 2518 | 2027 | 933 | 2518 |
| Lateral I | L1 | 4160 | 2250 | 2210 | 1976 | 886 | 2210 |
| Lateral I | L2 | 3588 | 1889 | 2001 | 1905 | 784 | 2001 |
| Lateral I | L3 | 3089 | 1793 | 1582 | 1733 | 912 | 1582 |
| Lateral I | L4 | 2018 | 1401 | - | 1451 | - | - |
| Lateral I | L5 | 1675 | 1158 | - | 655 | - | - |
| Lateral I | L6 | 1241 | - | - | 871 | - | - |
| Lateral II | L7 | 1039 | - | - | 688 | - | - |
| Lateral II | L8 | 1110 | - | - | 597 | - | - |
| Lateral II | L9 | 894 | - | - | 505 | - | - |
| Tip | T1 | 735 | - | - | 430 | - | - |

Supplementary Table S1.

The leading edge cross section dimensions for the Cicada 1 wing. Hereafter, the sample numbers are in the ascending order within each wing section in the direction from the wing root to the wing tip.

| Wing section | Sample  number | Costa outer *D_co_*, *μm* | Costa inner *D_ci_*, *μm* | Costa wall thickness, *T_c_, μm* | Subcosta outer *D_so_*, *μm* | Subosta inner *D_si_*, *μm* | Subcosta wall thickness, *T_s_*,  *μm* |
| --- | --- | --- | --- | --- | --- | --- | --- |
|
| Root | R1 | 2716 | 1472 | 238 | 2527 | 2368 | 207 |
| Root | R2 | 2592 | 1435 | 156 | 2536 | 1831 | 267 |
| Root | R3 | 2644 | 1566 | 147 | 2284 | 1895 | 227 |
| Medial | M1 | 2353 | 1784 | 119 | 2425 | 2032 | 175 |
| Medial | M2 | 2412 | 1756 | 120 | 2355 | 1956 | 142 |
| Medial | M3 | 2354 | 1850 | 131 | 2394 | 2075 | 171 |
| Medial | M4 | 2341 | 1732 | 178 | 2317 | 2138 | 165 |
| Medial | M5 | 2353 | 1749 | 122 | 2358 | 1983 | 155 |
| Medial | M6 | 2328 | 1763 | 187 | 2205 | 1999 | 136 |
| Lateral I | L1 | 2212 | 1801 | 140 | 2091 | 1868 | 120 |
| Lateral I | L2 | 1989 | 1665 | 125 | 1887 | 1708 | 87 |
| Lateral I | L3 | 1827 | 1585 | 138 | 1475 | 1273 | 94 |
| Lateral I | L4 | 1487 | 1281 | 157 | - | - | - |
| Lateral I | L5 | 1674 | - | 46 | - | - | - |
| Lateral I | L6 | 1310 | - | 102 | - | - | - |
| Lateral II | L7 | 1125 | - | 97 | - | - | - |
| Lateral II | L8 | 1054 | - | 123 | - | - | - |
| Lateral II | L9 | 821 | - | 88 | - | - | - |
| Tip | T1 | 686 | - | 66 | - | - | - |

Supplementary Table S2.

Other dimensions for the Cicada 1 wing cross sections.

| Cicada | Wing section | Sample  number | Cross section total height,  *H_t_*, *μm* | Costa height, *H_c_*,*μm* | Costa tip height, *H_ct_*, *μm* | Costa width, *W_c_*, *μm* | Costa tip width, *W_ct_*, *μm* | Subcosta width, *W_s_*, *μm* |
| --- | --- | --- | --- | --- | --- | --- | --- | --- |
| 2 | Root | R1 | 3201 | 1786 | 889 | 1518 | 484 | 1825 |
|  | Root | R2 | 2663 | 1578 | 789 | 1373 | 569 | 1937 |
|  | Root | R3 | 2470 | 1397 | 595 | 1339 | 623 | 1876 |
|  | Root | R4 | 2506 | 1393 | 686 | 1270 | 686 | 1914 |
|  | Medial | M1 | 2422 | 1179 | 534 | 1285 | 711 | 1812 |
|  | Medial | M2 | 2273 | 1176 | 545 | 1332 | 742 | 1772 |
|  | Medial | M3 | 2316 | 1124 | 537 | 1292 | 754 | 1776 |
|  | Medial | M4 | 2322 | 1182 | 560 | 1318 | 759 | 1764 |
|  | Medial | M5 | 2232 | 1148 | 512 | 1353 | 694 | 1727 |
|  | Medial | M6 | 2175 | 1112 | 469 | 1381 | 728 | 1375 |
|  | Medial | M7 | 1976 | 917 | 482 | 1430 | 724 | 1592 |
|  | Lateral | L1 | 1788 | 887 | 433 | 1401 | 734 | 1440 |
|  | Lateral | L2 | 1619 | 1178 | - | 1157 | - | - |
|  | Lateral | L3 | 1156 | - | - | 850 | - | - |
|  | Lateral | L4 | 889 | - | - | 698 | - | - |
|  | Lateral | L5 | 895 | - | - | 623 | - | - |
|  | Lateral | L6 | 838 | - | - | 544 | - | - |
|  | Tip | T1 | 647 | - | - | 384 | - | - |
|  | Tip | T2 | 626 | - | - | 334 | - | - |
|  | | | | | | | | |
| 3 | Medial | M1 | 2659 | 1422 | 308 | 1059 | 469 | 1518 |
|  | Medial | M2 | 2533 | 1461 | 272 | 1091 | 448 | 1526 |
|  | Medial | M3 | 2522 | 1361 | 282 | 1150 | 504 | 1561 |
|  | Medial | M4 | 2464 | 1308 | 304 | 1098 | 547 | 1519 |
|  | Medial | M5 | 2380 | 1233 | - | 1088 | - | 1415 |
|  | Medial | M6 | 2373 | 1176 | - | 1084 | - | 1295 |
|  | Lateral | L1 | 2314 | 1246 | - | 1059 | - | 1319 |
|  | Lateral | L2 | 2646 | 1257 | - | 1062 | - | 1085 |
|  | Lateral | L3 | 964 | - | - | 593 | - | - |
|  | Lateral | L4 | 845 | - | - | 606 | - | - |
|  | Tip | T1 | 942 | - | - | 501 | - | - |
|  | Tip | T2 | 806 | - | - | 473 | - | - |
|  | Tip | T3 | 795 | - | - | 366 | - | - |
|  | Tip | T4 | 790 | - | - | 359 | - | - |

Supplementary Table S3.

The leading edge cross section dimensions for the wings of Cicadas 2 and 3.

| Cicada | Wing section | Sample  number | Costa outer *D_co_*, *μm* | Costa inner *D_ci_*, *μm* | Costa wall thickness, *T_c_, μm* | Subcosta outer *D_so_*, *μm* | Subosta inner *D_si_*, *μm* | Subcosta wall thickness, *T_s_*,  *μm* |
| --- | --- | --- | --- | --- | --- | --- | --- | --- |
| 2 | Root | R1 | 1819 | 1426 | 150 | 1686 | 1336 | 177 |
|  | Root | R2 | 1628 | 1388 | 124 | 1664 | 1272 | 167 |
|  | Root | R3 | 1523 | 1271 | 112 | 1573 | 1289 | 190 |
|  | Root | R4 | 1504 | 1191 | 156 | 1606 | 1247 | 144 |
|  | Medial | M1 | 1381 | 1189 | 129 | 1595 | 1308 | 139 |
|  | Medial | M2 | 1416 | 1194 | 123 | 1524 | 1246 | 148 |
|  | Medial | M3 | 1337 | 1136 | 93 | 1544 | 1336 | 146 |
|  | Medial | M4 | 1358 | 1136 | 119 | 1510 | 1308 | 151 |
|  | Medial | M5 | 1342 | 1127 | 134 | 1523 | 1206 | 193 |
|  | Medial | M6 | 1342 | 1138 | 107 | 1523 | 1220 | 142 |
|  | Medial | M7 | 1308 | 1110 | 105 | 1365 | 1201 | 140 |
|  | Lateral | L1 | 1306 | 1086 | 113 | 1241 | 1049 | 141 |
|  | Lateral | L2 | 1143 | 923 | 136 | - | - | - |
|  | Lateral | L3 | 1014 | 1025 | 116 | - | - | - |
|  | Lateral | L4 | 994 | 789 | 88 | - | - | - |
|  | Lateral | L5 | 914 | 751 | 77 | - | - | - |
|  | Lateral | L6 | 830 | - | - | - | - | - |
|  | Tip | T1 | 674 | - | - | - | - | - |
|  | Tip | T2 | 590 | - | - | - | - | - |
|  | | | | | | | | |
| 3 | Medial | M1 | 1272 | 1147 | 76 | 1360 | 1145 | 85 |
|  | Medial | M2 | 1316 | 1173 | 90 | 1339 | 1138 | 93 |
|  | Medial | M3 | 1262 | 1010 | 108 | 1433 | 1133 | 128 |
|  | Medial | M4 | 1229 | 1035 | 85 | 1347 | 1149 | 93 |
|  | Medial | M5 | 1166 | 972 | 72 | 1299 | 1152 | 77 |
|  | Medial | M6 | 1127 | 923 | 111 | 1253 | 1046 | 118 |
|  | Lateral | L1 | 1165 | 964 | 99 | 1185 | 1023 | 62 |
|  | Lateral | L2 | 1135 | 941 | 104 | 1287 | 1012 | 114 |
|  | Lateral | L3 | 887 | 747 | 92 | - | - | - |
|  | Lateral | L4 | 819 | 697 | 77 | - | - | - |
|  | Tip | T1 | 765 | - | - | - | - | - |
|  | Tip | T2 | 672 | - | - | - | - | - |
|  | Tip | T3 | 660 | - | - | - | - | - |
|  | Tip | T4 | 623 | - | - | - | - | - |

Supplementary Table S4.

Other dimensions for the wing cross sections of Cicadas 2 and 3.
